# Supplementary material for: Patterns of Intron Gain and Loss in Fungi
Source: PLoS Biol. 2004 Nov 30;2(12):e422. doi: 10.1371/journal.pbio.0020422 (PMC532390; doi:10.1371/journal.pbio.0020422)
Supplement: Table S1 — Also available at http://genes.mit.edu/NielsenEtAl/. (4.3 MB ZIP). [file pbio.0020422.st001.zip › NielsenEtAl/html/434.html]

AN5338.1.NCU07332.1.MG05574.1.FG06539.1


```
 CLUSTAL W (1.82) Multiple Sequence Alignments - Introns Inserted


Sequence 1: MG05574.1	523 aa
Sequence 2: FG06539.1	515 aa
Sequence 3: NCU07332.1	538 aa
Sequence 4: AN5338.1	519 aa
Alignment Length: 553 aa
Number Identitical Residues: 204 aa
Alignment Score (without introns) 11786


MG05574.1 	--MSKSKSADDSDMSRPGDIAVLPLQGAFQIYQTLADRFLSPSPPKD---IPKDAP--TI
NCU07332.1	-------MTLESRVQNP---ILQPLLFIFQVIQFVLNKLLSPTPPSP--KARLGRP--RI
FG06539.1 	-------MPFNSGLVNP---ILHPLIFLFQIFQWITAKLLSPRPPNENARLGHNRP--KI
AN5338.1  	MPKSSSQSSLSTFARQP---FLQTFTFFYVIFQNILNWIFAPGPPPS-GPIANNLPKKRV
          	 . :.:. . .:   .*    : .:   : : * :   :::* **   .    . *.. :

MG05574.1 	AIIGAGITGVTAAAHCVGHGFNVVIFEAGSRRN-LGGIWT~AVNDSSSLQIHSAMYRFHP
NCU07332.1	AVIGGGITGVTSAAHCVGHGCDVRIFEAGKEEN-IGGIWS0RVNNTSGLQIHSLMYRFHP
FG06539.1 	AVIGAGITGVTSAAHCIGHGFDVVIFEAGSKDN-VGGIWS0RVNETSGLQIHSLMYRFHP
AN5338.1  	AIIGTGLTGVSSAAHCVGHGFDVQLFEKRSKEKGLGGIWS0RVNSTSSLQIHSIMYRFHP
          	*:** *:***::****:*** :* :**  .. :.:****:  **.:*.***** ******

MG05574.1 	SIRWSRSYPDRKEILDQVEKLWWRYGLDKVTKFKVRVKRVYQD-DDGRWIVNH--PEHGK
NCU07332.1	SVNWKQGYPDKQQILSQVRELWKRYRLDERTVFETPVTSARQHPETKKWIINNDAETYGQ
FG06539.1 	SVQWNRGYPDRQQILSQVRHLWERYGLKERTRFNTKIDRVYQD-EQGRWIVNN--PSLGR
AN5338.1  	SVHYDAAYPTQEEIRDQIVDLWKRYKLEDRTVFETPVESVKQT-RTGKWIINNDEEKWGL
          	*:.:. .** :::* .*: .** ** *.. * *:. :  . *     :**:*:.    * 

MG05574.1 	FEGLITAVGTCGKPKIPIFPDREKFKGPVYHSSQLNG2KDAAGKRIAIIGGGASAVEVLE
NCU07332.1	FDGIICAIGTCGRPKIPHIPGMDSFQGEIHHSSQLTG~KAAKGKTVIIIGGGASAVESIE
FG06539.1 	FGGVIAAVGTCGAPKMPKIHGMDNFKGPIYHSSDLTG2KDVKGKKMAIIGGGASAVESLE
AN5338.1  	FDGVVASVGVCGDPKMISLPDQTNFKGSVYHSSELDG~KQVKGKKVLIIGGGASAIEALE
          	* *:: ::*.** **:  : .  .*:* ::***:* * * . ** : ********:* :*

MG05574.1 	WAFKGGAAHASILSRSEKWIIPRNIVINTLLSWNVLGQDAFIGWLPEILLRRFFYRDLDS
NCU07332.1	YAVEEGAAQVYVLARSEKWIIPRNPVVDMLLALNIFGQETRFSWIPEFFLRKLFYRELED
FG06539.1 	FAFAEEAAQVSILSRSDKWIIPRNMFVDALLSFNIFGHETVLSFIPEFFLRKFFYRDLEN
AN5338.1  	FAVKSGASEIDVLSRSDKWIIPRNVWVQSLLALNIFGQETFLSWIPESLLHKFFYRDLQD
          	:*.   *:.  :*:**:*******  :: **: *::*::: :.::** :*:::***:*:.

MG05574.1 	IAPS--NKGIFTDTPMVNSDVLDHIRSGKADWVRCDVESLTDSGVLVNERAPGVPKGGLG
NCU07332.1	IAPPSGKKGLFTDTPMVNSEVMNQIRSGKVKWVRCDIDGFTENGVKINRREKGTNPKDAG
FG06539.1 	ISPAK-NHGLFMETPMVNSDIMDKLREGKVEWVRCDIDEFVYQGVRVNRRAKGVPQGGPG
AN5338.1  	IAPSG---GLYTQTPMANSELFDQIREGKARWLRGDILSVKEEGILFNKRSKGVPQGGPG
          	*:*.    *:: :***.**::::::*.**. *:* *:  .  .*: .*.*  *.   . *

MG05574.1 	RPRVVDADIIVMATGFKRPSLAFLPDDCFNDPYGPPNWYLQTFPPTHPSISAINC2TYIS
NCU07332.1	QTEKIKGDMVIMATGFQRPKLDFLPKECLEGDYKPPNWYLQTFPPQFPTVAAINC~TYMN
FG06539.1 	HEEVIDADIIVMATGYKRPSLSFLPDDCFREPYPPPNWYLQTFPPSHPSVSAINC~TYVN
AN5338.1  	RETVIPGDVIIQATGFKRPSLAFLPEDAFEEPYGPPSWYLQVFPPSYPSICANNS~TYVN
          	:   : .*::: ***::**.* ***.:.:.  * **.****.*** .*::.* *. **:.

MG05574.1 	AIGTVGNWHIGIYTRILLMFLTDPLTRPAPWWMRRWIDMTKLLKATSPVGAFDFFTYLEL
NCU07332.1	GIGSVGNWHIGIYTRLLLVFLLDDRARPRTFWMKRWIDMTAFLKRRSPVGPFEFFTYLEL
FG06539.1 	AIGTVGNWHIGIYTRILLMFLMDPLSRPSPFWMQRWIDLTKTLKLTSPTGAFDFFTYLEL
AN5338.1  	AIGTVGNMHIGIYTRFLLMFLVDPLSRPTEGRMKTWIDFTRFMKRLSPVGAFDFFTYAEL
          	.**:*** *******:**:** *  :**    *: ***:*  :*  **.*.*:**** **

MG05574.1 	LWWFLFCICFNPFRWKWALFVFLGLGLGLPKETTELETRLLQRAGWDRREVGRDVGKSI-
NCU07332.1	MWWFFFCVAINPFRWKWAFFVFFGLGENLPMKVVAAEERLRSRLQAREAEDQGSMAKGVR
FG06539.1 	LWWFFFCVVVNPFRWKWAIFVFLGVGFGLLEAVVSQEERITNGNGYHAR----DEGTSL-
AN5338.1  	IYWFVFVILVNPFRWKWAPFVLFGIGRGLPAQVVKQEEAFRNALRKQYAT----------
          	::**.* : .******** **::*:* .*   ..  *  : .                  

MG05574.1 	----------------
NCU07332.1	HVREEEIRGRTMRIVT
FG06539.1 	----------------
AN5338.1  	----------------
          	
```
